# Factors associated with demand for emergency medical services by people with hypertension and diabetes

**DOI:** 10.1590/0034-7167-2022-0147

**Published:** 2023-05-08

**Authors:** Patrícia Chatalov Ferreira, Sonia Silva Marcon, Elen Ferraz Teston, Viviane Cazetta de Lima Vieira, Rebeca Rosa de Souza, Mislaine Casagrande de Lima Lopes, Verônica Francisqueti Marquete, Robson Marcelo Rossi

**Affiliations:** I Universidade Estadual de Maringá. Maringá, Paraná, Brazil; II Universidade Federal de Mato Grosso do Sul. Campo Grande, Mato Grosso do Sul, Brazil

**Keywords:** Hypertension, Diabetes *Mellitus*, Electronic Health Records, Emergency Medical Services, Primary Health Care

## Abstract

**Objectives:**

to analyze the association between recurrence of emergency service visits due to lack of blood pressure and/or glycemic control with sociodemographic variables and disease registration in Primary Care.

**Methods:**

quantitative study, which consulted medical records of people who attended these services two or more times for 26 months. Descriptive statistics and multiple logistic regression models were used in analysis.

**Results:**

most people did not have hypertension and/or diabetes record in their Primary Care records. The absence of this record was more frequent in males, aged between 18 and 59 years, with low education and lack of blood pressure. There was association between greater number of people seeking these services in the same year and not monitoring the chronic condition in specialized care.

**Conclusions:**

people who do not follow up hypertension and/or diabetes in Primary Care are more likely to need assistance due to blood pressure and/or glycemic management.

## INTRODUCTION

The organization of services that make up the Unified Health System (SUS - *Sistema Único de Saúde*) in Brazil aimed to guarantee the right to health and continuity of care at different points of care, since operating in isolation, regardless of the service’s level of complexity, does not allow for implementing SUS principles and guidelines. Thus, for people to receive comprehensive, continuous and quality care, it is necessary to integrate the different services and professionals who work in them as well as the establishment of common care flows^(1)^.

Among the points that make up the Health Care Network (RAS - *Rede de Atenção à Saúde*), it is defined that Primary Health Care (PHC) is the gateway and the axis of communication with all other service points, interconnecting services from lower to higher technological density^(2)^. In this context, the emergency room (ER) is part of the Emergency Care Network and is in charge, 24 hours a day, of the care of different health conditions: acute or chronic, acute, clinical, surgical, traumatological, among others^(3)^.

In a systematic review with meta-analysis, the authors identified that hypertensive conditions in ER services are prevalent, persistent and predictive of cardiovascular outcomes in the long term, and these, in turn, are the leading causes of premature death in the western world. They concluded that outpatient follow-up of these patients is essential for complication prevention and postponement^(4)^.

Many consultations carried out in ER services, related to acute health conditions that, in principle, could be treated in PHC^(5)^, result from the lack/deficiency of interaction between the different RAS service points. Thus, high rates of Hospitalizations due to Primary Care-Sensitive Conditions (HPCSC) may indicate serious problems with access to the health system or its performance^(6)^.

Diseases, including arterial hypertension (AH), angina pectoris, heart failure and diabetes mellitus (DM) are part of the HPCSC list^(6)^. National studies found the persistence of a high proportion of expenses attributed to cardiovascular diseases and the significant increase in hospitalizations for angina^(7-8)^, whose main factors for its development are AH and DM^(9)^.

In Brazil, ischemic heart disease and stroke have been the main causes of death and years lived with disability since the late 1960s^(10)^. Therefore, adequate monitoring and control of the causes of these chronic conditions in PHC can prevent progression and the emergence of complications, in addition to minimizing the number of hospitalizations and cardiovascular mortality^(11)^.

In order to help fight chronic diseases and non-communicable diseases, a strategic action plan for Brazil was prepared, effective for 2021 to 2030. This plan aims to expand PHC coverage with screening, identification, management and follow-up services for people with AH and DM, by encouraging the qualification of clinical and care work by health professionals and implementation of lines of care^(12)^.

Some studies point out flaws in screening and follow-up of people with AH and DM by PHC teams^(5,13-14)^. As a result, in the management of chronic conditions, only half or a third of people are diagnosed, and, of these, half or a third have this condition under control and are enrolled in effective programs for disease promotion or prevention^(15)^. Other studies point out that older adults and females access PHC services more frequently, while men, especially those of working age, are regular users of ER services. Thus, recognizing the importance of PHC actions in preventing complications of AH and DM, the following questions were elaborated: do people who recurrently seek ER services due to acute complications of these diseases have their diagnosis registered in PHC? What are the sociodemographic and health monitoring characteristics of people with and without registration of these conditions in PHC who seek ER services due to uncontrolled AH and/or DM?

## OBJECTIVES

To analyze the association between recurrence of emergency service visits due to lack of blood pressure and/or glycemic control with sociodemographic variables and disease registration in Primary Care.

## METHODS

### Ethical aspects

To carry out this study, the determinations recommended by Resolutions 466/2012 and 510/2016 of the Brazilian National Health Council were complied with. The project was approved by the signatory institution’s Permanent Research Ethics Committee. In compliance with the General Data Protection Law (Law 13.709/18) provisions, specific care was taken with the database that contained data on participants, especially in relation to their identification.

### Study design, place and period

This is a descriptive, cross-sectional study with a quantitative approach, extracted from a master’s thesis, defended in 2020, entitled “Acute complications in people with Hypertension and Diabetes mellitus: subsidies for screening and monitoring in the Health Care Network”. The STrengthening the Reporting of OBservational studies in Epidemiology (STROBE) recommendations guided the preparation of its report.

The study was carried out in a municipality in northwestern Paraná, using the electronic medical records of the Health Department and University Hospital (UH) care network as a data source. This municipality has an estimated population of 423,666 people and coverage of 85% by the Family Health Strategy (FHS) teams. The public RAS is made up of 34 Basic Health Units (BHU), a municipal hospital, two municipal Emergency Care Units (ECU), ER UH, a polyclinic and a Intermunicipal Public Health Consortium (CISAMUSEP - *Consórcio Público Intermunicipal de Saúde*) specialized outpatient clinic and a UH.

Data were collected from December 2019 to March 2020. For this purpose, the systems of each of the three ER services were consulted, identifying all the people who sought them out due to blood pressure or glycemic control in 2018 and 2019 and in January and February 2020. In one of the units, data were collected only from 2019 onwards, when the electronic medical record was implemented.

In this survey, sequential filters were used, such as year, ICD-10, age and date of search. AH-related ICDs were: I10, I15, I15.9, I11, I11.0, I11.9, I12, I12.0, I12.9, I13, I13.0, I13.1, I13.2, I13.9, G45, G45.8, I64, I21, I21.0, I21.1, I21.2, I21.3, I21.4, and I21.9, O11, I20, I20.8, I20.9. For the DM-tracer condition, the ICDs were: E10 to E10.9, E11 to E11-9, E13 to E13-, E14 to E14.9, N08.3, O24.0 to O24.3, E16.0 to E16,2, G59.0, G63.2, H28.0, H36.0, M14.2 and R73.9.

### Population and sample; inclusion and exclusion criteria

The study population consisted of people who sought one of the three ER services in the city during the period considered for the study, due to conditions related to acute complications of AH and/or DM. In February 2020, there were 36,658 people registered in the electronic medical record for AH and 11,857 for DM, and in the period under study, 7,632 people sought previously defined ER services due to causes/ICD, which were responsible for 10,649 consultations.

Individuals aged 18 years or older, residing in the municipality or in one of its two districts and having sought the ER service twice or more during the study period for causes related to AH and/or DM were included. Individuals who do not reside in the municipality, died or presented incomplete registration were excluded.

### Study protocol

Initially, a survey was carried out of all searches for the service motivated by defined causes, manually preparing a single list of all visits in the three services, and, based on the name, date of birth and medical record number, people who sought the service twice or more were identified. Considering the exclusions (48 deaths, five incomplete data, 134 residents in other municipalities), a sample of 1,182 people was obtained, corresponding to 3,209 admissions.

Subsequently, the management system used by the Municipal Health Department (MHD) and the UH management system were accessed, consulting the electronic medical records of 1,182 people for registration data collection, such as date of birth, BHU of reference, family situation, education, skin color and marital status.

Follow-up in the CISAMUSEP specialized care network (yes/no) was identified from a nominal list made available by the service of all people who underwent consultation with cardiologist, endocrinologist and/or with a member of the multidisciplinary team (nurse, pharmacist, physical educator, nutritionist and occupational therapist) during the study period.

### Analysis of results, and statistics

Data were stored in a Microsoft Office Excel 2021^®^ spreadsheet and transferred to the R^(16)^ program for processing and analysis. For analysis, the multiple logistic regression model^(17-18)^ was used via the GLM command (Generalized Linear Models) and the Forward-Backward Stepwise method (MASS library), which allowed the best fit determination using the Akaike criterion (AIC).

The measure of association between independent variables (sex, skin color, age, education, marital status, family arrangement, service sought, reason for admission, number of admissions, follow-up in specialized care at CISAMUSEP or outpatient clinic at the UH and BHU certified by the Qualification Program for Primary Health Care - APSUS (*Qualificação da Atenção Primária à Saúde*)) and outcome variable has AH and/or DM registered in PHC’s electronic medical record, which was determined by the Odds Ratio (OR) and respective 95% confidence intervals. To check the final adjusted regression model quality, the Hosmer and Lemeshow (H-L) test^(18)^ was used, in addition to measuring the area under the ROC curve (AUC)^(19)^ and graphically verifying residues’ behavior via a simulated binomial envelope^(19)^.

## RESULTS

Of the 1,182 people with two or more admissions to ER services due to acute events resulting from AH and/or DM, more than half (55.1%) did not have any of the conditions registered in PHC’s electronic medical record. Of those who had a record, 52.4% had a record of AH; 36.5% of AH and DM and 11.1% of DM.

Table 1 shows that most of these people lived with someone (95.2%), had low education (71.4%), white skin color (70.1%), partner (60.4 %), were female (56.9%), and aged 60 years or older (52.8%). In adjusted analysis, it was identified that men were approximately twice as likely to not have a registered condition compared to women. The same occurred with adults compared to older adults (four times more likely) and with those without a partner (1.34 times more likely). A higher level of education was a protective factor, as the chance of not having any condition registered in PHC electronic medical record was twice as high for people with low education.

**Table 1:** (Adjusted) multivariable Odds Ratio for independent sociodemographic variables of people who have or do not have a record of hypertension and diabetes mellitus in the electronic medical record of a municipality in southern Brazil, 2021

Variables	Has a diagnosis of AH and/or DM registered with PHC						
	Yes	No	Total	Adjusted final model			
	n (%)	n (%)	n (%)	*p* value	OR	CI(OR, 95%)	
Sex (n=1,182)							
Female	331 (49.3)	341 (50.7)	672 (56.9)	0.00123	1.56	1.19	2.05
Male	200 (39.2)	310 (60.8)	510 (43.1)				
Age (years) (n=1,182)							
18├ ┤59	149 (26.7)	409 (73.3)	558 (47.2)	<0.001	3.74	2.85	4.93
60├	382 (61.2)	242 (38.8)	624 (52.8)				
Color (n=1,167)							
White	381 (46.58)	437 (53.4)	818 (70.1)	Ns			
Non-white	148 (42.41)	201 (57.6)	349 (29.9)				
Marital status - with partner (n=1,158)							
Yes	338 (48.4)	361 (51.6)	699 (60.4)	0.03469	1.34	1.02	1.76
No	192 (41.8)	267 (58.2)	459 (36.6)				
Family arrangement - living with someone (n=1,158)							
Yes	500 (45.4)	602 (54.6)	1102 (95.2)	Ns			
No	30 (53.6)	26 (46.4)	56 (4.8)				
Education (n=1,118)							
≤8 years	419 (52.5)	379 (47.5)	798 (71.4)	0.00165	0.61	0.44	0.82
> 8 years	95 (29.7)	225 (70.3)	320 (28.6)				

H-L test: p-value = 0.074; AUC ROC = 0.739;

* Ns - not significant.

Table 2 contains consultation characteristics in the researched ER services and follow-ups in PHC and specialized care.

**Table 2:** (Adjusted) multivariate Odds Ratio for independent variables of use of health services by people who have or do not have a record of hypertension and diabetes mellitus in Primary Care’s electronic medical record in a municipality in southern Brazil, 2021

Variables	Has a diagnosis of AH and/or DM registered with PHC						
	Yes	No	Total	Adjusted final model			
	n (%)	n (%)	n (%)	*p* value	OR	CI(OR, 95%)	
Search reason (n=1182)							
Uncontrolled high blood pressure	342 (40.2)	508 (59.8)	850 (71.9)	0.0006	2.2	1.41	3.50
Uncontrolled glycemic control	120 (55.5)	100 (45.5)	220 (18.6)	0.6178	Ns		
Both the conditions	69 (61.6)	43 (38.4)	112 (9.5)				
Service searched for (n=1182)							
ECU	470 (43.8)	603 (56.2)	1073 (90.8)				
ER UH	11 (61.1)	7 (39.9)	18 (1.5)	Ns			
Both services	50 (54.9)	41 (45.1)	91 (7.7)	Ns			
Recurrence of admission/demand for emergency services (n=1,182)							
One admission in years ≠	139 (53.3)	122 (46.7)	261 (22.1)				
2 or more in the same year	392 (42.6)	529 (57.4)	921 (77.9)	0.0022	1.63	1.21	2.24
Number of admissions (n=1,182)							
2 admissions	329 (43.9)	420 (56.1)	749 (63.4)	Ns			
3 to 5 admissions	179 (46.3)	208 (53.7)	387 (32.7)	Ns			
> 5 admissions	23 (50.0)	23 (50.0)	46 (3.9)				
Specialized care follow-up at CISAMUSEP (n=1,182)							
Yes	30 (83.3)	6 (16.7)	36 (3.1)				
No	501 (43.7)	645 (56.3)	1146 (96.9)	0.0012	5.39	2.11	16.8
Specialized care follow-up - University Hospital Outpatient Clinic (n=1,182)							
Yes	7 (87.5)	1 (12.5)	8 (0.7)				
No	524 (44.6)	650 (55.4)	1174 (99.3)	Ns			
APSUS certified reference BHU (n=1,182)							
Yes	270 (49.1)	280 (50.9)	550 (46.5)				
No	261 (41.3)	371 (58.7)	632 (53.5)	Ns			

* H-L test: p-value = 0.074. AUC ROC = 0.739;

* Ns - not significant; CISAMUSEP - Intermunicipal Public Health Consortium; APSUS - Primary Health Care Qualification Program; BHU - Basic Health Unit.

There was a predominance of admissions related to AH and/or complications (71.9%), followed by DM and/or complications (18.6%) and by both conditions (9.5%). Attendances were concentrated in the ECU (90.8%). People who sought ER UH (61.1%) or both services (54.9%) presented condition registration in PHC. There was a higher proportion of condition registrations in PHC among people who sought ER services only once in the same year (53.3%), while two or more searches in the same year were more frequent among people who had no registration (57.4%).

The proportion of users who underwent specialized care at CISAMUSEP (3.1%) or at the UH outpatient clinic (0.7%) was small, but most of them had some of the listed conditions (83.3% AH and 87.5% % diabetes). Furthermore, no significant difference was observed in relation to whether or not the reference BHU was certified by the APSUS.

In the multivariate analysis (Table 2), the results indicated that people who sought ER services due to AH and/or complications were 2.2 times more likely to have no registered chronic condition compared to users who sought these services for both morbidities. People with two or more admissions in any of the years were 1.63 times more likely to not be registered with PHC. Finally, people who did not undergo specialized assistance had 5.39 more chances of not having the registration of these conditions.

## DISCUSSION

Initially, this study showed that, although more than half of those who sought ER services two or more times were female and over 60 years old, the greatest chances of not having either of the two health conditions, in the study, registered in PHC’s medical record, were observed in males, aged between 18 and 59 years and up to eight years of study. The first two characteristics are already highlighted in the literature as factors associated with lower use and accessibility to primary health services^(13-14,20-23)^.

With regard to sex, men’s preferential demand for ER services is influenced by cultural and social issues that involve the determinants of the health-disease process and permeate the use of health services^(13,24)^. The higher proportion of AH and/or DM diagnosis registration in women can be explained by the fact that they are more diligent about symptoms, seek greater knowledge about the condition and attend health units more^(21,25)^. It should be noted that, in ER services, it is common for men to be accompanied by their wives, who are often the ones who report their partner’s complaints, highlighting their role as caregivers within the family nucleus^(26)^.

Men, in turn, generally seek health services when they already have symptoms and, sometimes, clinical urgency, as identified in a population-based study carried out with 410 men aged 20 to 59 years in a municipality in northwestern Paraná^(27)^ and in Campina Grande, Paraíba^(28)^. For this reason, they prioritize immediate care and greater accessibility offered by ER units^(28)^, which justifies the greater chance of men not having a record of AH and/or DM diagnosis at the primary level.

The lack of registration of AH and/or DM in individuals aged 18 to 59 years may be due to limited access to primary services, since care at this level of care mostly occurs during business hours, coinciding with the work shift of a large portion of the population of working age^(14,29)^. In this way, ER services become easily accessible health units, often used by this group^(30)^.

Some strategies may favor linking this public with health services and, therefore, following up their health condition, especially those who already live with a chronic disease. Among these strategies are the expansion of service hours for the working public, such as the *Programa Saúde na Hora* ^(31)^, and carrying out health promotion actions in partnership with companies registered in the area covered by the unit.

The fact of finding that people without a partner had a greater chance of not having registered with PHC allows us to infer that this presence favors follow-up and coping with chronic conditions, but its absence can contribute to the adoption of unhealthy habits and even neglect of one’s own health^(26,32)^. Hence, a study carried out with men, in the countryside of São Paulo, identified that the association between the presence of AH, a smaller number of friends and a low perception of social support^(33)^ was more frequent among those who lived alone and/or who did not have a partner.

The family’s role with members affected by chronic conditions is relevant, encouraging or supporting them in the adoption of self-care actions and better disease monitoring in PHC. Therefore, the mapping of families in the area covered by the health unit^(34)^ allows professionals, especially the nursing team, to identify opportunities to work with other members of the family nucleus, aiming to sensitize them about their role in the process of monitoring and appropriate management of chronic conditions.

It can also be said that the finding that a higher educational level is a protective factor in relation to chronic condition registration in PHC’s medical record. This can be attributed to the idea that higher education favors access to information about the disease, generates more knowledge about the clinical status itself and strategies indicated for proper management and, as a consequence, greater possibility of adopting healthier behaviors, in addition to raising awareness of the implications that the absence of such care can generate^(20,23)^.

In addition to the factors that were shown to be related to whether or not the chronic condition was registered in PHC, and considering that only cases with two or more admissions were analyzed, the number of people who sought care at ER services for acute events resulting from uncontrolled blood pressure and/or blood sugar levels is noteworthy. However, this is not a situation experienced in isolation in the municipality under study or in the country. The assessment of adults with AH and/or DM in a municipality in northern Paraná found that 30.8% of them did not have a record of their health condition in the basic units, although they were followed up by the FHS teams^(27)^. Internationally, a study carried out in northern Tanzania also found a high frequency of cases of uncontrolled AH and DM in ER services, and the complications of these conditions were responsible for more than a quarter of all hospitalizations of adults^(35)^.

Certainly, the fact of having the condition registered in PHC would not avoid all cases of demand for ER services due to acute conditions, but this number could be minimized through adequate follow-up, especially in cases of recurrent demand. Furthermore, this register is essential for the effective monitoring of AH and DM in the different points of care of the RAS and the offer of actions that are more consistent with the population’s real health needs^(36)^. Therefore, reliable knowledge of the population assigned and affected by these conditions is paramount^(37)^, which can be operationalized through territorialization associated with risk stratification.

In the literature, there are two types of factors that can trigger inconsistency in the records of people with AH and DM and, therefore, make it difficult and/or prevent the full monitoring and follow-up of the affected population. Among the factors related to the organization of services, there are the health units, with overcrowded areas, a very high number of demands, productivity requirements for the entire team, especially for Community Health Workers (CHW), and lack of preparation or overload of PHC professionals in the face of these obstacles. Regarding users, the factors involved are not knowing the diagnosis, knowing but not admitting/accepting that they have it, not adhering to the recommended/prescribed treatment, or believing that they are already cured^(38)^.

Users’ lack of knowledge about the diagnosis of a chronic condition, as evidenced in CHW reports, in a study carried out in Florianópolis, Santa Catarina^(38)^, may justify the fact that people who sought BP services due to lack of blood pressure were twice as likely to not have the condition registered in PHC. It should be noted that this disease usually evolves slowly and is often silent or asymptomatic, and sporadic manifestations of symptoms are not interpreted as a disease and/or complication. Therefore, there is no recognition of the need to change habits and monitor the condition in primary health services^(5,14,38)^.

Registering the health condition, in addition to being an important report, makes it easier for the team to establish a minimum criterion, if there is a need to refer users to specialized care, aiming at comprehensive and continuous care and greater effectiveness in the assistance offered^(25-28)^. A study that assessed the trend of hospitalization and death due to ischemic stroke in Brazil over a period of 15 years found that, shortly after implementing the Hypertensive and Diabetic Registration and Monitoring System (HIPERDIA) in 2002, there was a decline of more than 70% of cases^(39)^.

The reason for the absence of this register, especially in cases in which the demand for ER services was recurrent, perhaps it was due to non-appreciation/recognition of the seriousness of the situation or due to some difficulty in accessing the service, whether personal or related to the care network organization/structure. Regardless of the reason, these people are not being adequately assisted in PHC, which makes it impossible to prevent new events. Lack of knowledge about their real health condition and the absence of follow-up by a multidisciplinary team lead to acute events and serious health complications that can impact quality of life^(40)^. One cannot fail to consider that the organization and access to services influence continuity of care in PHC^(25-28)^. The difficulty of accessing health units, in addition to being a barrier to managing the health condition, may also be responsible for the lack of diagnosis and, consequently, early management, which increases the risk of complications^(41-42)^.

The Brazilian Ministry of Health establishes how to identify people with AH and DM and how to adequately follow them up at different levels of care. In PHC, for instance, it is proposed, among others, using the following indicators: proportion of people residing in the assigned area of the unit registered with a diagnosis of AH or DM; average attendance for these groups; and proportion of people with these conditions followed up at home^(43)^.

The longitudinal assessment of indicators can support planning, management and assessment of policies and preventive and assistance actions for these people, and at the same time allows identifying and assessing inequalities in the provision of services aimed at these groups. Failure to use the proposed indicators, or unsatisfactory results, may mean poor monitoring and poor control of these conditions in PHC, favoring the emergence and progression of these diseases, increasing the number of hospitalizations and deaths from these causes^(42)^.

The analysis of the production of 112 FHS teams in Florianópolis, Santa Catarina, for instance, pointed out that, despite the increase in registration indicators, there was no increase, in the same proportion, in monitoring indicators^(44)^. In Cambé, Paraná, the number of home visits, nursing and medical consultations to groups at high cardiovascular risk did not meet the standard of care established^(27)^. A study that assessed the health care of people with DM, from the Chronic Care Model’s (CCM) perspective, found that the actions were developed with a biomedical and curative focus, i.e., with a great distance from what is proposed by the model^(45)^. The low compliance of care practices, according to cardiovascular risk stratification and metabolic control, impacting the quality of care offered, was also identified in a survey carried out in the state of Minas Gerais with 108 older adults with AH and/or DM being followed up at PHC^(46)^.

It is urgent to raise awareness of FHS teams about the importance of registration and the territorialization of the assigned area, the development of intersectoral actions and social control actions of users with AH and/or DM, aiming to articulate the health care offered with the health needs of the population living in its coverage area, according to the different risk strata^(46)^. Furthermore, the logistical system of communication, between the different points of the network, needs special attention from managers, in order to develop strategies to face this barrier in the continuity of care, an essential attribute of PHC.

In this context, the importance of nurses stands out, who, in fact, exercise the role of team coordinator, even informally, in addition to being a key element in the implementation of actions and care programs developed in PHC. Effective monitoring and management of people with AH and/or DM, using strategies such as active search, health education groups, telemonitoring, home visits and reception with active listening can contribute to the control of exacerbations of signs and symptoms, guiding the therapy and care plan review for this group of users^(34)^.

In the municipality under study, health actions, shared by a multidisciplinary team, aiming at comprehensiveness and resolvability of care, occur with the support of specialized assistance, both at CISAMUSEP and at the UH outpatient clinic. However, the operating systems used in these units are different and not linked to those of the Municipal Health Department (MHD). The absence of a unified information system contributes to the fragmentation of care for people with chronic conditions and triggers failure in follow-up and monitoring in the RAS.

The operating system integration would allow professionals to access users’ medical records from any point in the network. In addition, the system could be improved so that the reference BHU was communicated every time a user sought a ER service due to blood pressure and/or glycemic control. Health teams would find it easier, through an active search, to approach users with uncontrolled health conditions, more effectively monitor the chronic condition and plan BHU actions and routines^(34,38,47)^.

The CCM implementation is under development, and only BHU with gold, silver and some bronze seals have already carried out the stratification of users in their enrollment area and can refer high-risk users to specialized care. The other BHU need to be able to follow up by adapting to users in chronic conditions. Probably, for this reason, people who never received specialized care were five times more likely to have no registered chronic condition.

The results found evidence the fragmentation and disarticulation of the different points in the RAS, more specifically between PHC and ER services. Therefore, it is urgent to implement actions that favor the articulation between the different services that make up the RAS so that the demands met in ER services are known by PHC teams. The existence of a referral program for urgent and emergency services for preventive outpatient care was pointed out, in the Tanzanian study, as an effective way of linking people to PHC^(35)^.

It is essential, therefore, that public policies consider the need to improve the connection between the different levels of complexity and human health, point out mechanisms that favor their accessibility to PHC health services^(28)^ and enable the proposition of specific actions and interventions that encourage the adoption of preventive practices and greater use of this level of care^(22-23)^.

### Study limitations

Possible limitations of this study are inherent to those that make use of secondary data, as there is a possibility of failure to record information, outdated records, under-identification of people who sought the service at least twice, considering that the electronic medical record in one of the ECU was implemented only in January 2019. There is still a lack of knowledge about the characteristics of adherence to the treatment proposed within the scope of PHC and also about people who do not use the public system to monitor their health condition, but do so when they need emergency care.

### Contributions to nursing and health

Considering that nurses are the main organizer of actions within the scope of PHC, especially in the management of practices for monitoring NCDs, the results of this study are relevant to the area, showing that people with AH and DM often seek ER services as a result of changes in blood pressure and blood sugar levels and that many of these people do not have a record of the chronic condition in PHC.

## CONCLUSIONS

More than half of the people who sought the city’s ER services two or more times due to acute events of AH and/or DM did not have their chronic condition registered in PHC’s electronic medical record. The characteristics that remained associated with greater chances of not being included in this register were being male, age in the productive phase - from 18 to 59 years old -, low education and demand because of uncontrolled blood pressure. The lack of this record was associated with the following consequences: higher frequency of two or more admissions in some of the years surveyed and non-referral to specialized care.
